# Morphological Adaptation of Cave-Dwelling Ground Beetles in China Revealed by Geometric Morphometry (Coleoptera, Carabidae, Trechini)

**DOI:** 10.3390/insects12111002

**Published:** 2021-11-08

**Authors:** Mengzhen Chen, Wanru Guo, Sunbin Huang, Xiaozhu Luo, Mingyi Tian, Weixin Liu

**Affiliations:** 1 Department of Entomology, College of Plant Protection, South China Agricultural University, 483 Wushan Road, Guangzhou 510642, China; 18819427325@163.com (M.C.); g1597667714@163.com (W.G.); huangsunbin@163.com (S.H.); xiaozhu.luo@uni-jena.de (X.L.); 2 Mécanismes Adaptatifs et Évolution (MECADEV), Muséum National d’Histoire Naturelle, CP50, 57 Rue Cuvier, 75005 Paris, France; 3 Institute of Zoology and Evolutionary Research, Friedrich Schiller University Jena, Erbertstr. 1, 07743 Jena, Germany

**Keywords:** adaptive type, cave environment, morphological variation, phenotypic development

## Abstract

**Simple Summary:**

Cavernicolous ground beetles dwelling in China are one of the most diverse and underground-adapted coleopteran group in the world. The tribe Trechini is, among them, the most representative group constituting over 170 known species with a narrow and elongated body and long appendages or a stout body and short appendages. However, very little information about their morphology has been explored. The aim of this study was to analyze the morphological adaptations of this group using geometric morphological methods. The beetles were divided into four different morphological types, including aphaenopsian, semi-aphaenopsian, anophthalmic, and surface-dwelling, and the analysis is based on the morphology of their head, pronotum, and elytra. Our findings indicate that the overall morphological variation of cave trechine beetles has gradually specialized from an anophthalmic to semi-aphaenopsian to aphaenopsian type. Different types have different directions of variation in the head, pronotum, and elytra, but the pronotum is more differentiated and morphologically diverse than the head and elytra.

**Abstract:**

Cave-dwelling ground beetles in China represent the most impressive specific diversity and morphological adaptations of the cavernicolous ground beetles in the world, but they have not been systematically examined in quantitative terms. The present study focuses on the application of geometric morphological methods to address the morphological adaptations of the tribe Trechini, the most representative group in China. We have employed a geometric morphometry analysis of the head, pronotum, and elytra of 53 genera of Trechini, including 132 hypogean and 8 epigean species. Our results showed that the overall morphological variation of cave carabids has gradually specialized from an anophthalmic to semi-aphaenopsian to aphaenopsian type. There were extremely significant differences (*p* < 0.01) among four different adaptive types including aphaenopsian, semi-aphaenopsian, anophthalmic, and surface-dwelling Trechini when their adaptability to a cave environment was used as the basis for grouping. Furthermore, there were differences in the phenotypic tree of the head, pronotum, and elytra, and an integrated morphology. To the best of our knowledge, this is the first report on the analysis of the head, pronotum, and elytra of four different adaptive types of ground beetles in order to clarify the morphological adaptations of cavernicolous carabids to the cave environment.

## 1. Introduction

**Figure 1:**
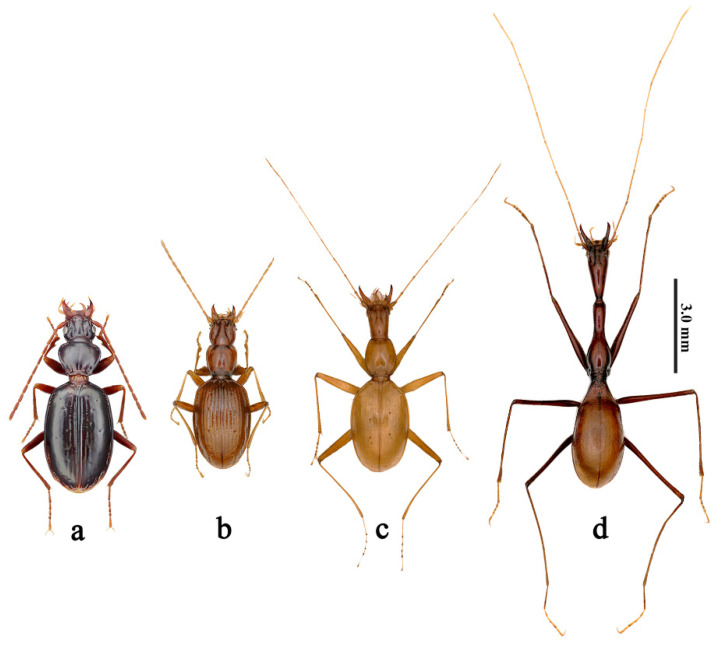
Morphological characteristics of four different adaptive types of ground beetles. (**a**) Surface-dwelling (*Sinotrechiama yunnanus*); (**b**) anophthalmic (*Sinotroglodytes yanwangi*); (**c**) semi-aphaenopsian (*Aspidaphaenops dudou*); and (**d**) aphaenopsian (*Giraffaphaenops clarkei*).

China is very rich in cave-dwelling ground beetles. At present, 202 species of cave carabids belonging to 8 tribes and 71 genera have been recorded, among which the Trechini is the most diverse group including 175 species from 63 genera [1,2]. All of them are troglobionts and completely lack eyes, except for four troglophiles with more or less degenerated eyes [3]. In addition to the disappearance of their eyes, cavernicolous carabids underwent morphological modifications during long-term adaptation to the subterranean environment. These are manifested in the loss of pigment and metathoracic wings, as well as their more slender body and thin legs [4,5]. According to the adaptability of characteristic appearances and biological information, cave trechine beetles were divided into three morphological types [6,7,8]: aphaenopsian, semi-aphaenopisan, and anophthalmic (Figure 1). The former means that these carabids have an extreme elongation of their body and appendages, while the latter refers to their stout body and shorter appendages. The semi-aphaenopsian is considered to be a transitional type, with morphological characteristics lying between the above two. The surface-dwelling trechine beetles have darker body color and compound eyes (Figure 1).

Geometric morphometry is an approach that relies on the quantitative analysis of the geometry of the target structure and the further performance of statistical analyses [9]. Different types of data, such as landmark coordinates, outline curves, and surfaces are used to define the shape [10]. The original morphological information is usually obtained through Cartesian coordinates, which are used to remove the interference of non-morphological variation in the analysis so that the punctuation overprint analysis of all samples can be visualized and displayed [11]. Geometric morphometry began to be used in the 1980s, and in the 21st century it has been widely used in entomology, medicine, archaeology, and other fields [12,13,14]. Recently, geometric morphometry has developed from two-dimensional to three-dimensional. Three-dimensional scanning, electron microscope scanning, micro-CT scanning, etc., provide advanced technical support for the development of geometric morphometry [15,16,17].

The application of geometric morphometry to coleopteran insects is very extensive. It is often used to explore morphological differences between species with sexual dimorphism [18,19], intersubspecies, sister groups [20,21], and high-level categories [22,23]. It is also possible to infer the ancestral form of an existing taxa in order to study its origin and evolution [24]. Geometric morphometry has gradually been applied to different groups of Coleoptera, including Carabidae [25,26,27], Lucanidae, Chrysomelidae [28], Scarabaeidae [29,30,31], and Silphidae [32]. However, studies of the geometric morphometry of cave-dwelling ground beetles have rarely been reported [33].

Based on a geometric morphometric approach, the present paper provides, for the first time, an analysis of the head, pronotum, and elytra of four different adaptive types of ground beetles in order to clarify the morphological adaptations of cavernicolous carabids to the cave environment. In addition, the phenotypic relationship was obtained with a clustering analysis in the genetic category to explore the morphological evolution of cave-dwelling ground beetles.

## 2. Materials and Methods

### 2.1. Studied Materials

For the materials used in this study, we implemented the following principles: (1) Sampling as many genera and species as possible, including type species. (2) We did not deal with subgenus separately; species taxonomic treatment was based on the latest publications. (3) We used bibliographic data to obtain the morphological adaptation types of known taxa, e.g., [34,35,36,37,38].

A total of 140 species in 53 genera of Trechini (49 genera and 132 species from caves and 4 genera and 8 species of surface-dwelling beings) were examined in this study (Table A1). Among them, 97 specimens were deposited at South China Agricultural University, e.g., [39,40,41].

### 2.2. Geometric Morphometric Approach

#### 2.2.1. Image Acquisition

Photographs of existing samples were taken with a Keyence VHX-5000 digital microscope (Figure S1). Due to the lack of specimens for 44 species, images were obtained from related original literature, e.g., [42,43,44] (Figure S1). For *Minimaphaenops* (*Enshiaphaenops*) *senecali* Deuve, 2016, we gathered the data from the specimen we collected as well as the additional figure of the type specimen from the original publication. After editing using Adobe Photoshop CS6, we imported the data into tps-Util 1.78 [45].

#### 2.2.2. Landmark Data

**Figure 2:**
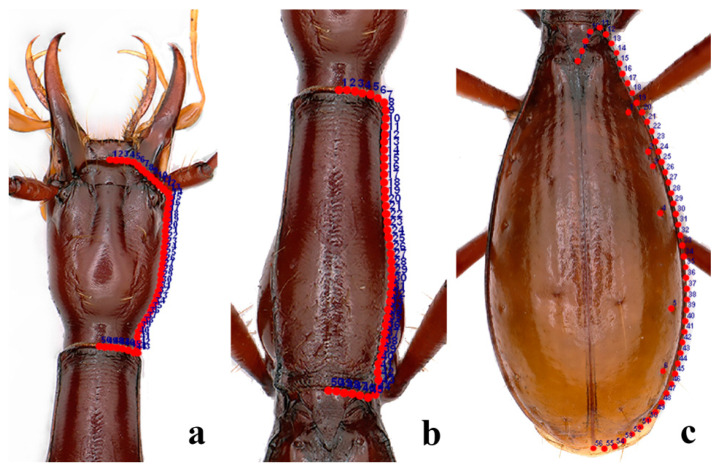
Landmark and semilandmark configurations of ground beetle specimens. (**a**) The right side of the head (50 positions); (**b**) right side of the pronotum (50 positions); and (**c**) right side of the elytra (56 positions).

The shape of the head, pronotum, and elytra and the positions of stable pores on the elytra were chosen as morphological indicators. We selected the configurations of 50 semilandmarks of each object, except for the stable pores on the elytra, which were represented by 6 landmarks (Figure 2). The landmarks and semilandmarks in each image were digitized using the tps-Dig 2.31 software (Landmark Data S1). The Tps-Small 1.34 software [46] was used to detect the data correlation of the.tps files after the landmarks to verify whether the data correlation met the requirements.

#### 2.2.3. Statistical Analysis

The morphological data obtained by the standardized processing of different cave environment-adapted types were imported into the MorphoJ 1.07A software. Generalized Procrustes Analysis (GPA) was used to perform Procrustes superimposition on the overall samples to extract shape variables [47,48]. We used calculational processing to ensure that the sum of the squares of the distances between landmarks of the same serial number was minimized. Additionally, we calculated the overall average shape to compare the degree of difference between the individual and overall average shape (measured by Procrustes distance). On this basis, we applied principal component analysis (PCA).

We selected the first two principal components (PC) to construct scatterplots to show the morphological differences of carabids in different cave environment adaptation types. An energy map of the extreme points of the coordinate origin arrangement was obtained from a thin-plate spline (TPS) analysis using the MorphoJ 1.07A software, where differences in the landmarks were displayed in a visual form.

On the basis of PCA, we set the different cave environment adaptation types as the basis for grouping and performed canonical variate analysis (CVA). The results were displayed through the Mahalanobis distance and Procrustes distance.

#### 2.2.4. Clustering Analysis

The original TPS file was split into 53 subfiles according to genera using the tps-Util 1.78 software; then we used the tps-Super 2.05 software to calculate the average form of each genus. Procrustes distances between genera were preformed using the tps-Small 1.34 software; then we used the unweighted group averaging method (UPGMA) in the NTSYSpc 2.10e software [49] to analyze the Procrustes distance matrix.

## 3. Results

### 3.1. Internal Correlation of the Original Data

The original data were converted from camber Kendall space to Euclidean tangent space. For the head, pronotum, and elytra, the correlation coefficients of the data before and after the conversion were 0.999995, 0.999992, and 1.000000, respectively, which met the requirements.

### 3.2. PCA of the Morphological Variation in Head, Pronotum and Elytra

**Figure 3:**
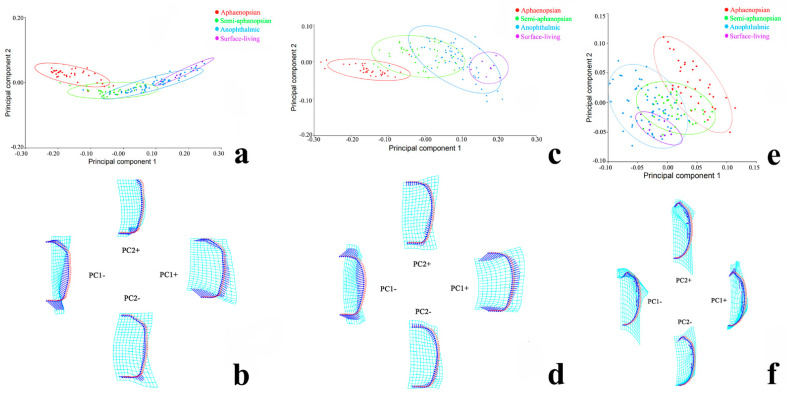
Principal component analysis (PCA) of four different adaptive types of ground beetles: (**a**,**b**) head; (**c**,**d**) pronotum; (**e**,**f**) elytra. (**a**,**c**,**e**) represent scatterplot; (**b**,**d**,**f**) represent the energy map.

Principal component analysis was performed on the morphological data of the head, pronotum, and elytra for 141 species of carabid beetles, with 96, 96, and 108 principal components being obtained, respectively. Among them, the first principal component (PC1) accounted for 89.86%, 84.95%, and 39.35% of the overall variance, while the second principal component (PC2) accounted for 3.94%, 7.75%, and 27.20%, respectively. Using PC1 and PC2, which affect the morphological variation, as the abscissa and ordinate, respectively, a scatterplot of the morphological variation was obtained, and a 90% equal frequency ellipse was constructed based on the cave adaptation type of these carabid beetles (Figure 3a,c,e).

From the perspective of the morphological variation of the head (Figure 3a) and pronotum (Figure 3c), it was found that the aphaenopsian had no overlap and could be distinguished well from anophthalmic and surface-dwelling carabid beetles. The semi-aphaenopsian carabid beetle type was between the aphaenopsian and anophthalmic types and was more similar to the anophthalmic type because of its larger overlaps. The latter was closer to the surface-dwelling carabid beetles. Regarding the morphological variation of the elytra (Figure 3e), these four types overlapped more overall. No significant differences were found between elytra of the semi-aphaenopsian, aphaenopsian, and anophthalmic types.

The energy map of the coordinate origin and the extreme points of the PCA scatterplot of carabid beetles’ head, pronotum, and elytra (Figure 3b,d,f) show that the length/width ratio of the head and pronotum has a tendency to decrease significantly, while their lateral edges expand outward in the positive direction of PC1. The widest point of the head moves to the front, but the front edge of the pronotum tends to be wider than the rear edge. Elytra tend to be more slender and the scutellum appears to be narrower. The anterior of the edge side of the elytra has a tendency to undergo adduction, with the shoulders disappearing and the position of the hair pores moving to the distal end of the elytra. In the positive direction of PC2, the posterior edge of the head is sunken inward and the front and caudal corners of the pronotum tend to become acute from an obtuse angle. The first four elytra hair pores are more scattered and the last three are closer together.

### 3.3. CVA of the Morphological Variation in Head, Pronotum, and Elytra

According to the results of the PCA, CVA was used to analyze the morphological variation in the distances of the head, pronotum, and elytra among all the genera of carabid beetles. The results showed that the Mahalanobis distance and Procrustes distance between aphaenopsian and surface-dwelling types were largest when adaptability to a cave environment was used as the basis for grouping. For the head, pronotum, and elytra, the maximum Mahalanobis distance (Table 1) was 28.5719, 20.8313, and 20.7926, respectively, while the maximum Procrustes distance (Table 2) was 0.3429, 0.3258, and 0.1032, respectively.

The Mahalanobis distance (Table 3) and Procrustes distance (Table 3) of the head, pronotum, and elytra were tested to determine the significance of the differences. It was shown that the four different types (aphaenopsian, semi-aphaenopsian, anophthalmic, and surface-dwelling carabid beetles) had high significant differences from each other (*p* < 0.01).

### 3.4. The Phenotypic Evolutionary Relationship between Cave Trechini Genera

**Figure 4:**
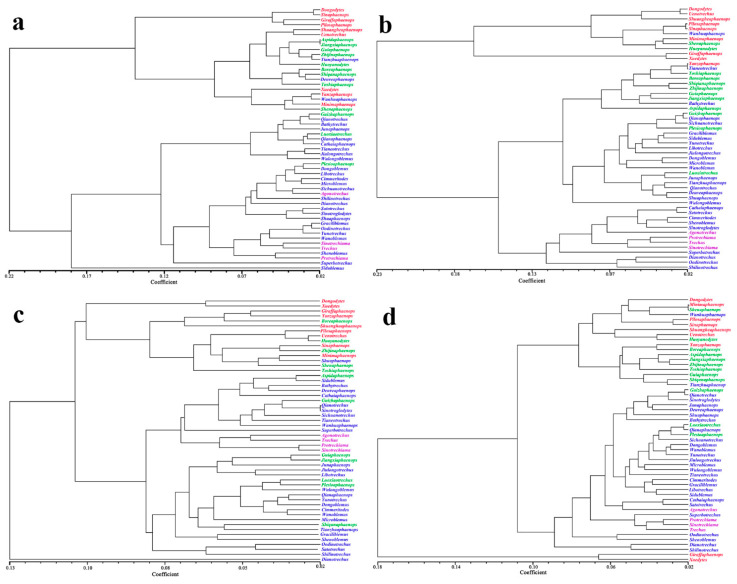
Phenotypic tree of ground beetle genera based on the Procrustes distance. (**a**) Head, (**b**) pronotum, (**c**) elytra, and (**d**) integrated morphology. Anphaenopsian: red letters; semi-anphaenopsian: green letters; anophthalmic: blue letters; surface-dwelling: purple letters.

Based on the Procrustes distance matrix of the average morphology among 53 genera of carabid beetles, a cluster analysis was performed to construct a morphological phenotypic tree, including the head, pronotum, elytra, and all three (Figure 4). The results revealed that there were differences between the four phenotypic trees, but the variation trend of the head and the integrated morphological phenotypic tree was relatively close. When the 53 genera branched for the first time, the aphaenopsian and semi-aphaenopsian genera clustered into a clade, while the anophthalmic and surface-dwelling genera of carabid beetles clustered into another clade.

In the head phenotypic tree (Figure 4a), *Sidublemus* was the first to be differentiated into a single branch. *Dongodytes*, *Sinaphaenops*, *Giraffaphaenops*, and *Pilosaphaenops* were all found to be closely related. However, *Shuangheaphaenops*, *Uenotrechus*, *Xuedytes*, *Yanzaphaenops*, and *Minimaphaenops* were mixed together with mostly semi-aphaenopsian genera carabid beetles. From the integrated phenotypic tree (Figure 4d), it could be seen that *Giraffaphaenops* and *Xuedytes* were the earliest to differentiate, and they were located far from other genera. The relationship between *Yanzaphaenops* of the aphaenopsian and semi-aphaenopsian group was relatively close, while *Wanhuaphaenops* of the anophthalmic group had a close relationship to those of the aphaenopsian group.

In the pronotum phenotypic tree (Figure 4b), all the aphaenopsian group except for *Yanzaphaenops* was combined into a clade with *Wanhuaphaenops*, *Shenaphaenops*, and *Huoyanodytes*. The remaining three types of carabids genera were clustered together, while about 1/3 of the anophthalmic type were grouped with surface-dwelling carabid beetles in another clade. The result of the elytra phenotypic tree (Figure 4c) showed that *Dianotrechus* was the first to be differentiated into a single branch. The aphaenopsian group and a small part of the semi-aphaenopsian group were clustered into a clade. Among them, *Dongodytes* is closely related to *Xuedytes*, but the same highly specialized *Sinaphaenops* was far away from the other genera in the aphaenopsian group. Part of the semi-aphaenopsian and anophthalmic groups were grouped together with surface-dwelling carabids.

## 4. Discussion

### 4.1. Morphological Variation Direction of Cave-Adapted Trechine Beetles

The highly specialized morphological characteristics of cave-dwelling ground beetles have long attracted the attention of researchers [50]. Most previous studies in this area have focused on changes in the morphology of cave-dwelling ground beetles after their long-term adaptation to cave life [51,52,53]. The present research is the first to attempt to study the morphological adaptation and variation direction of cave-dwelling ground beetles using geometric morphological analysis.

In the extreme environment of caves, animals often show the adaptive characteristics of convergent evolution due to environmental pressure [54,55]. Luo et al. [56,57] found that the cave-dwelling *S. wangorum* shows a distinct head posterior constriction and elongated pronotum combined with long and slender legs. Our results showed that the more slender their body is the higher the degree to which the ground beetles had adapted to the cave environment. This is mainly manifested in the fact that the widest point of the head gradually moves to the front, while the anterior edge of the pronotum tends to be narrower than the posterior edge in surface-dwelling compared to aphaenopsian carabids. Surprisingly, there is little available information concerning the elytra vitiation of cavernicolous carabids or other beetles [58]. We found that the position of the hair pores gradually moved towards the edge of the elytra, except for the scutellum, with the elytra becoming slenderer in cave carabids. One of the reasons why elytra is slenderer is a consequence of reducing or the disappearance of hind wings (also known as humeral calli) [59], and this situation is more distinct among the cave-dwelling species, especially the highly specialized ones.

In addition, aphaenopsian species mostly wander on stalactite walls or roofs in complete darkness [60], while semi-aphaenopsian species run on low rock walls or along the ground [61]. Anophthalmic species often live under small rocks or under damp dead wood in caves, and their habits are relatively close to those of surface-dwelling species [62,63,64]. It is speculated that the extension of the head and pronotum of cave-adapted ground beetles effectively increases the flexibility of the head, which may help this species to find prey in caves where food is scarce [65]. In contrast, surface-dwelling carabids may face great survival challenges [66,67] and their strong bodies will help them to fight and escape when faced with threats.

### 4.2. Geometric Morphology Analysis to Judge the Phylogeny of Cavernicolous Carabids

The molecular phylogeny of cave Trechini in China was analyzed based on two mitochondrial and two nuclear genes [68]. The preliminary study showed that the Chinese cave Trechini of Carabidae does not form a monophyletic lineage but rather is composed of four main independent evolutionary clades, each of which contains at least one highly convergent troglomorphic species.

In our study, certain differences exist in the morphological phenotypic trees of the head, pronotum, and elytra based on the Procrustes distance of carabids. For example, typical aphaenopsian genera—such as *Dongodytes*, *Giraffaphaenops*, *Sinaphaenops*, and *Xuedytes*—show extreme morphological specialization, but they are not clustered into same clade phylogenetically (Figure 4) [69]. Moreover, the semi-aphaenopsian genera of *Shenaphaenops* and *Huoyanodytes* and the anophthalmic type of *Wanhuaphaenops* are more closely related to the aphaenopsian type. A similar situation was also found in the Pyrenean subterranean Trechini, where the phylogenetic relationship between species of the same morphological type was not found to be close [70]. It may therefore be inferred that various Trechini lineages were settled multiple times independently in caves and underwent parallel morphological changes.

Furthermore, we did not classify the subgenus as an independent taxon in the present study, but the morphological differences between some subgenera in the same genus are relatively considerable. These differences may have a certain impact on the results of overall average shape.

Moreover, the length between the clade of *Giraffaphaenops* + *Xuedytes* and other genera is extensive in the integrated morphological phenotypic tree (Figure 4). This may have been caused by long periods of geographical isolation, or there may still be large gaps between these two genera and others. Future geometric morphometry of research for these groups could focus on adding the missing new genera and combining molecular phylogeny and biogeography for analysis.

## Figures and Tables

**Table 1:** Mahalanobis distances from four adaptive types of ground beetles based on the head, pronotum, and elytra, respectively.

	Aphaenopsian	Semi-Aphaenopsian	Anophthalmic
Semi-aphaenopsian	11.1596//8.9433//12.6030		
Anophthalmic	13.9436//10.7282//15.3457	6.6324//7.6456//7.0232	
Surface-dwelling	28.5719//20.8313//20.7926	23.0163//16.3761//18.8595	22.5005//16.7463//16.2172

**Table 2:** Procrustes distances from four adaptive types of ground beetles based on the head, pronotum, and elytra, respectively.

	Aphaenopsian	Semi-Aphaenopsian	Anophthalmic
Semi-aphaenopsian	0.1319//0.1409//0.0516		
Anophthalmic	0.2451//0.2342//0.0854	0.1227//0.0995//0.0485	
Surface-dwelling	0.3429//0.3258//0.1032	0.2275//0.1981//0.0644	0.109//0.101//0.0589

**Table 3:** *P*-values of the differences in Mahalanobis and Procrustes distances for the four adaptive types of ground beetles (10,000 permutation test, consistent for the head, pronotum, and elytra).

	Aphaenopsian	Semi-Aphaenopsian	Anophthalmic
Semi-aphaenopsian	<0.0001		
Anophthalmic	<0.0001	<0.0001	
Surface-dwelling	<0.0001	<0.0001	<0.0001

## Data Availability

Not applicable.

## Supplementary Materials

The following are available online at https://www.mdpi.com/article/10.3390/insects12111002/s1, Figure S1: Photographs of samples, Landmark Data S1: Semi-landmarks of samples.

## Appendix A

**Table A1:** Basal information of the studied specimens used in the geometric morphometric analysis.

No.	Species	Locality	Adaptive Type
1	*Dongodytes* (*Dongodytes*) *baxian* Tian, 2011	Guangxi, Du’an County	aphaenopsian
2	*D.* (*D.*) *elongatus* Tian, Yin & Huang, 2014	Guangxi, Du’an County	aphaenopsian
3	*D.* (*D.*) *fowleri* Deuve, 1993	Guangxi, Bama County	aphaenopsian
4	*D.* (*D.*) *giraffa* Uéno, 2005	Guangxi, Tian’e County	aphaenopsian
5	*D.* (*D.*) *grandis* Uéno, 1998	Guangxi, Fengshan County	aphaenopsian
6	*D.* (*D.*) *lani* Tian, Yin & Huang, 2014	Guangxi, Du’an County	aphaenopsian
7	*D.* (*D.*) *tonywhitteni* Yang, Huang & Tian, 2018	Guangxi, Bama County	aphaenopsian
8	*D.* (*D.*) *troglodytes* Tian, Yin & Huang, 2014	Guangxi, Du’an County	aphaenopsian
9	*D.* (*Dongodytodes*) *brevipenis* Tian, Yin & Huang, 2014	Guangxi, Du’an County	aphaenopsian
10	*D.* (*D.*) *deharvengi* Tian, 2011	Guangxi, Du’an County	aphaenopsian
11	*D.* (*D.*) *inexpectatus* Tian, Yin & Huang, 2014	Guangxi, Du’an County	aphaenopsian
12	*D.* (*D.*) *jinzhuensis* Tian, Yin & Huang, 2014	Guangxi, Du’an County	aphaenopsian
13	*D.* (*D.*) *yaophilus* Tian, Yin & Huang, 2014	Guangxi, Dahu County	aphaenopsian
14	*Giraffaphaenops clarkei* Deuve, 2002	Guangxi, Leye County	aphaenopsian
15	*G. yangi* Tian & Luo, 2015	Guangxi, Tianlin County	aphaenopsian
16	*M.* (*Enshiaphaenops*) *senecali* Deuve, 2016	Hubei, Enshi Autonomous Prefecture	aphaenopsian
17	*M.* (*Minimaphaenops*) *lipsae* Deuve, 1999	Chongqing, Fengjie County	aphaenopsian
18	*Pilosaphaenops pilosulus* Deuve & Tian, 2008	Guangxi, Huanjiang County	aphaenopsian
19	*P. whitteni* Tian, 2010	Guangxi, Huanjiang County	aphaenopsian
20	*Shuangheaphaenops elegans* Tian, 2017	Guizhou, Suiyang County	aphaenopsian
21	*Sinaphaenops* (*Dongaphaenops*) *xuxiakei* Deuve & Tian, 2014	Guizhou, Pan County	aphaenopsian
22	*S.* (*Sinaphaenops*) *banshanicus* Tian, Chen & Tang, 2017	Guizhou, Guiding County	aphaenopsian
23	*S.* (*S.*) *gracilior* Uéno & Ran, 1998	Guizhou, Libo County	aphaenopsian
24	*S.* (*S.*) *lipoi* Chen, Huang & Tian, 2020	Guizhou, Guiyang City	aphaenopsian
25	*S.* (*S.*) *mirabilissimus* Uéno & Wang, 1991	Guizhou, Libo County	aphaenopsian
26	*S.* (*S.*) *mochongensis* Tian & Huang, 2015	Guizhou, Duyun City	aphaenopsian
27	*S.* (*S.*) *orthogenys* Uéno, 2002	Guizhou, Sandu County	aphaenopsian
28	*S.* (*S.*) *wangorum* Uéno & Ran, 1998	Guizhou, Libo County	aphaenopsian
29	*S.* (*S.*) *yaolinensis* Tian, Chen & Yang, 2017	Guizhou, Duyun City	aphaenopsian
30	*S.* (*Thaumastaphaenops*) *bidraconis* Uéno, 2002	Guizhou, Ziyun County	aphaenopsian
31	*S.* (*T.*) *pulcherrimus* Magrini, Vanni & Zanon, 1997	Guizhou, Ziyun County	aphaenopsian
32	*Uenotrechus deuvei* Tian & Chen, 2017	Guangxi, Du’an County	aphaenopsian
33	*U. gejianbangi* Tian & Wei, 2017	Guangxi, Huanjiang County	aphaenopsian
34	*U. liboensis* Deuve & Tian, 1999	Guizhou, Maolan County	aphaenopsian
35	*U. nandanensis* Deuve & Tian, 2010	Guizhou, Nandan County	aphaenopsian
36	*Xuedytes bellus* Tian & Huang, 2017	Guangxi, Du’an County	aphaenopsian
37	*Yanzaphaenops hirundinis* Uéno, 2005	Hubei, Shennongjiia	aphaenopsian
38	*Aspidaphaenops dudou* Tian & Huang, 2018	Guizhou, Xingyi County	semi-aphaenopsian
39	*A. masakii* Uéno, 2006	Guizhou, Xingyi County	semi-aphaenopsian
40	*A. reflexus* Uéno, 2006	Guizhou, Ceheng County	semi-aphaenopsian
41	*A. volatidraconis* Uéno, 2006	Guizhou, Xingyi County	semi-aphaenopsian
42	*A. xiongda* Tian & Huang, 2018	Guizhou, Anlong County	semi-aphaenopsian
43	*Boreaphaenops angustus* Uéno, 2002	Hubei, Shennongjiia	semi-aphaenopsian
44	*Guiaphaenops deuvei* Tian, Feng & Wei, 2017	Guangxi, Lingyun County	semi-aphaenopsian
45	*G. lingyunensis* Deuve, 2002	Guangxi, Lingyun County	semi-aphaenopsian
46	*Guizhaphaenops* (*Guizhaphaenops*) *giganteus* Uéno, 2000	Guangxi, Shuicheng County	semi-aphaenopsian
47	*G.* (*G.*) *lipsorum* Deuve, 1999	Yunnan, Zhenxiong County	semi-aphaenopsian
48	*G.* (*G.*) *pouillyi* Deuve & Queinnec, 2014	Guizhou, Pan County	semi-aphaenopsian
49	*G.* (*G.*) *striatus* Uéno, 2000	Guizhou, Liupanshui City	semi-aphaenopsian
50	*G.* (*G.*) *zhijinensis* Uéno & Ran, 2004	Guizhou, Zijin County	semi-aphaenopsian
51	*G.* (*G.*) *zorzini* Vigna Taglianti, 1997	Guangxi, Shuicheng County	semi-aphaenopsian
52	*G.* (*Semiaphaenops*) *lipsorum zunyiensis* Deuve & Tian, 2018	Yunnan, Zhenxiong County	semi-aphaenopsian
53	*G.* (*S.*) *martii* Deuve, 2001	Yunnan, Zhenxiong County	semi-aphaenopsian
54	*G.* (*S.*) *yudongensis* Deuve & Tian, 2016	Yunnan, Zhenxiong County	semi-aphaenopsian
55	*Huoyanodytes tujiaphilus* Tian & Huang, 2016	Hunan, Longshan County	semi-aphaenopsian
56	*Jiangxiaphaenops longiceps* Uéno & Clarke, 2007	Jiangxi, Shangrao City	semi-aphaenopsian
57	*Luoxiaotrechus deuvei* Tian & Yin, 2013	Hunan, You County	semi-aphaenopsian
58	*L. yini* Tian & Huang, 2015	Jiangxi, Pingxiang City	semi-aphaenopsian
59	*Plesioaphaenops annae* Deuve & Tian, 2011	Guangxi, Longlin County	semi-aphaenopsian
60	*Shenaphaenops humeralis* Uéno, 1999	Guangxi, Shuicheng County	semi-aphaenopsian
61	*Shiqianaphaenops majusculus* Uéno, 1999	Guizhou, Shiqian County	semi-aphaenopsian
62	*Toshiaphaenops globipennis* Uéno, 1999	Hubei, Xianfeng County	semi-aphaenopsian
63	*T. ovicollis* Uéno, 1999	Hunan, Longshan County	semi-aphaenopsian
64	*Zhijinaphaenops gravidulus* Uéno & Ran, 2002	Guizhou, Zijin County	semi-aphaenopsian
65	*Z. haozhicus* Deuve & Tian, 2018	Guizhou, Zijin County	semi-aphaenopsian
66	*Z. jingliae* Deuve & Tian, 2015	Guizhou, Xifeng County	semi-aphaenopsian
67	*Z. lii* Uéno & Ran, 2002	Guizhou, Zijin County	semi-aphaenopsian
68	*Z. liuae* Deuve & Tian, 2015	Guizhou, Xifeng County	semi-aphaenopsian
69	*Z. multisetifer* Deuve & Tian, 2018	Guizhou, Bijie City	semi-aphaenopsian
70	*Z. pubescens* Uéno & Ran, 2002	Guizhou, Zijin County	semi-aphaenopsian
712	*Z. wenganicus* Deuve & Tian, 2018	Guizhou, Wengan County	semi-aphaenopsian
72	*Z. zunyicus* Deuve & Tian, 2018	Guizhou, Zhunyi City	semi-aphaenopsian
73	*Bathytrechus rueci* Uéno, 2005	Guangxi, Leye County	anophthalmic
74	*Cathaiaphaenops* (*Amygdalotrechus*) *amplipennis* Uéno, 2000	Hubei, Xianfeng County	anophthalmic
75	*C.* (*A.*) *chuandongziensis* Deuve, 1999	Hubei, Banqiao Town	anophthalmic
76	*C.* (*A.*) *cychroides* Deuve & Tian, 2016	Hubei, Lichuan City	anophthalmic
77	*C.* (*A.*) *draconis* Deuve, 1999	Chongqing, Fengjie County	anophthalmic
78	*C.* (*A.*) *enshiensis* Deuve & Tian, 2016	Hubei, Banqiao Town	anophthalmic
79	*C.* (*A.*) *lagredeae* Deuve, 2016	Hubei, Enshi Autonomous Prefecture	anophthalmic
80	*C.* (*A.*) *lynchae* Deuve & Tian, 2008	Hubei, Jianshi County	anophthalmic
81	*C.* (*A.*) *vignatagliantii* Deuve, 1999	Chongqing, Fengjie County	anophthalmic
82	*C.* (*Cathaiaphaenops*) *delprati* Deuve, 1996	Hunan, Longshan County	anophthalmic
83	*Cimmeritodes* (*Cimmeritodes*) *huangi* Deuve, 1996	Hunan, Longshan County	anophthalmic
84	*C.* (*Dianocimmerites*) *crassifemoralis* Deuve & Tian, 2016	Yunnan, Zhenxiong County	anophthalmic
85	*C.* (*Shimenrites*) *shimenensis* Deuve & Tian, 2017	Hunan, Shimen County	anophthalmic
86	*C.* (*Xiangcimmerites*) *zhongfangensis* Deuve & Tian, 2016	Hunan, Zhongfang County	anophthalmic
87	*C.* (*Zhecimmerites*) *parvus* Tian & Li, 2016	Anhui, Chaohu City	anophthalmic
88	*C.* (*Z.*) *zhejiangensis* Deuve & Tian, 2015	Zhejiang, Changshan County	anophthalmic
89	*Deuveaphaenops* (*Deuveaphaenops*) *qimenxicus* Tian & Huang, 2017	Chongqing, Wulong District	anophthalmic
90	*D.* (*Furongius*) *gelaophilus* Tian & Huang, 2017	Guizhou, Zhunyi City	anophthalmic
91	*Dianotrechus gueorguievi* Tian, 2016	Yunnan, Anning City	anophthalmic
92	*Dongoblemus kemadongicus* Deuve & Tian, 2016	Yunnan, Zhenxiong County	anophthalmic
93	*Graciliblemus lipingensis* Deuve & Tian, 2016	Guizhou, Liping County	anophthalmic
94	*Jiulongotrechus pubescens* Tian, Huang & Wang, 2015	Guizhou, Tongren City	anophthalmic
95	*Junaphaenops tumidipennis* Uéno, 1997	Yunnan, Kunming City	anophthalmic
96	*Libotrechus duanensis* Lin & Tian, 2014	Guangxi, Dahu County	anophthalmic
97	*L. nishikawai* Uéno, 1998	Guizhou, Libo County	anophthalmic
98	*Microblemus rieae* Uéno, 2007	Zhejiang, Jinghua City	anophthalmic
99	*Oodinotrechus* (*Oodinotrechus*) *kishimotoi* Uéno, 1998	Guizhou, Libo County	anophthalmic
100	*O.* (*O.*) *liyoubangi* Tian, 2014	Guangxi, Huanjiang County	anophthalmic
101	*O.* (*Pingleotrechus*) *yinae* Sun & Tian, 2015	Guangxi, Pingle County	anophthalmic
102	*Qianaphaenops* (*Qianaphaenops*) *emersoni* Tian & Clarke, 2012	Guizhou, Yanhe County	anophthalmic
103	*Q.* (*Q.*) *longicornis* Uéno, 2000	Guizhou, Fenggang County	anophthalmic
104	*Q.* (*Q.*) *pilosus* Uéno, 2000	Guizhou, Jiangkou County	anophthalmic
105	*Q.* (*Q.*) *rotundicollis* Uéno, 2000	Guizhou, Sinan County	anophthalmic
106	*Q.* (*Q.*) *tenuis* Uéno, 2000	Guizhou, Fenggang County	anophthalmic
107	*Q.* (*Qiandongaphaenops*) *variabilis* Tian, Huang & Wang, 2015	Guizhou, Cengong County	anophthalmic
108	*Q.* (*Tiankengius*) *xigouicus* Tian & Huang, 2018	Shanxi, Hanzhong City	anophthalmic
109	*Qianotrechus* (*Qianotrechus*) *fani* Uéno, 2003	Sichuan, Gulin County	anophthalmic
110	*Q.* (*Q.*) *laevis* Uéno, 2000	Guizhou, Zhengan County	anophthalmic
111	*Q.* (*Q.*) *magnicollis* Uéno, 2000	Guizhou, Suiyang County	anophthalmic
112	*Q.* (*Q.*) *tenuicollis cheni* Uéno, 2003	Guizhou, Suiyang County	anophthalmic
113	*Q.* (*Sanwangius*) *rowselli,* Tian & Chen, 2019	Chongqing, Wulong District	anophthalmic
114	*Satotrechus longlinensis* Deuve & Tian, 2011	Guangxi, Longlin County	anophthalmic
115	*S. rieae* Uéno, 2006	Guizhou, Anlong County	anophthalmic
116	*Shenoblemus minusculus* Tian & Fang, 2020	Anhui, Huangshan City	anophthalmic
117	*Shilinotrechus fusiformis* Uéno, 2003	Yunnan, Shilin County	anophthalmic
118	*S. intricatus* Huang & Tian, 2015	Yunnan, Yiliang County	anophthalmic
119	*Shuaphaenops parvicollis* Uéno, 1999	Chongqing, Jinfoshan	anophthalmic
120	*Sichuanotrechus albidraconis* Uéno, 2006	Sichuan, Jiangyou City	anophthalmic
121	*S. dakangensis* Huang & Tian, 2015	Sichuan, Jiangyou City	anophthalmic
122	*Sidublemus solidus* Tian & Yin, 2013	Hunan, Guidong County	anophthalmic
123	*Sinotroglodytes bedosae* Deuve, 1996	Hunan, Longshan County	anophthalmic
124	*S. hygrophilus* Uéno, 2009	Hunan, Sangzhi County	anophthalmic
125	*S. yanwangi* Huang, Tian & Faille, 2020	Hubei, Yichang City	anophthalmic
126	*Superbotrechus bennetti* Deuve & Tian, 2009	Hubei, Yichang City	anophthalmic
127	*Tianeotrechus trisetosus* Tian & Tang, 2016	Guangxi, Tian’e County	anophthalmic
128	*Tianzhuaphaenops jinshanensis* Zhao & Tian, 2016	Guizhou, Tianzhu County	anophthalmic
129	*Wanhuaphaenops zhangi* Tian & Wang, 2016	Hunan, Chenzhou City	anophthalmic
130	*Wanoblemus wui* Tian & Fang, 2016	Anhui, Xuancheng City	anophthalmic
131	*Wulongoblemus tsuiblemoides* Uéno, 2007	Zhejiang, Jiangshan City	anophthalmic
132	*Yunotrechus diannanensis* Tian & Huang, 2014	Yunnan, Maguan County	anophthalmic
133	*Agonotrechus spinangulus* Belousov, Kabak & Liang, 2019	Sichuan, Muli County	surface-dwelling
134	*Protrechiama crassipes* Uéno, 1997	Sichuan, Meigu County	surface-dwelling
135	*Sinotrechiama yunnanus* Belousov, Kabak & Liang, 2019	Yunnan, Dayao County	surface-dwelling
136	*Trechus aghiazicus* Belousov & Kabak, 2019	Xinjiang, Zhaosu County	surface-dwelling
137	*T. cratocephalus* Belousov & Kabak, 2019	Xinjiang, Zhaosu County	surface-dwelling
138	*T. saluki* Belousov & Kabak, 2019	Xinjiang, Xinyuan County	surface-dwelling
139	*T. torgaut* Belousov & Kabak, 2019	Xinjiang, Hejing County	surface-dwelling
140	*T. tsanmensis* Belousov & Kabak, 2019	Xinjiang, Xinyuan County	surface-dwelling
